# Modeling Xanthan Gum Foam’s Material Properties Using Machine Learning Methods

**DOI:** 10.3390/polym16060740

**Published:** 2024-03-08

**Authors:** Halime Ergün, Mehmet Emin Ergün

**Affiliations:** 1 Seydisehir Ahmet Cengiz Faculty of Engineering, Necmettin Erbakan University, Konya 42360, Turkey; hboztoprak@erbakan.edu.tr; 2 Akseki Vocational School, Alanya Alaaddin Keykubat University, Antalya 07630, Turkey

**Keywords:** xanthan gum, foam, cellulose, machine learning, generalized regression neural networks

## Abstract

Xanthan gum is commonly used in the pharmaceutical, cosmetic, and food industries. However, there have been no studies on utilizing this natural biopolymer as a foam material in the insulation and packaging sectors, which are large markets, or modeling it using an artificial neural network. In this study, foam material production was carried out in an oven using different ratios of cellulose fiber and xanthan gum in a 5% citric acid medium. As a result of the physical and mechanical experiments conducted, it was determined that xanthan gum had a greater impact on the properties of the foam material than cellulose. The densities of the produced foam materials ranged from 49.42 kg/m^3^ to 172.2 kg/m^3^. In addition, the compressive and flexural moduli were found to vary between 235.25 KPa and 1257.52 KPa and between 1939.76 KPa and 12,736.39 KPa, respectively. Five machine-learning-based methods (multiple linear regression, support vector machines, artificial neural networks, least squares methods, and generalized regression neural networks) were utilized to analyze the effects of the components used in the foam formulation. These models yielded accurate results without time, material, or cost losses, making the process more efficient. The models predicted the best results for density, compression modulus, and flexural modulus achieved in the experimental tests. The generalized regression neural network model yielded impressive results, with R^2^ values above 0.97, enabling the acquisition of more quantitative data with fewer experimental results.

## 1. Introduction

Polymer foams are lightweight materials consisting of air voids enclosed by a polymeric matrix, which can be produced using foam generators that utilize gas or liquid. By reducing the density of the material, the air voids decrease the amount of raw material required, resulting in a lower cost of the final product. Additionally, the void content can be controlled, enabling foam material to be produced at varying densities, making it suitable for use in various applications such as sports equipment, packaging, insulation, food, automotive, and furniture [1]. However, most foam materials are produced from fossil-fuel-based polymers, including polypropylene, polyethylene, polyurethane, and polyamide. These materials have a low biodegradability rate and limited recyclability, with only 9% recycled [2]. In order to tackle this issue, researchers have explored the utilization of renewable resources in foam production, with a focus on materials that are sustainable, biodegradable, and recyclable [3]. Several natural polymers, such as chitosan [4], starch [5], alginate [6], and cellulose [7], have been utilized for this purpose. Cellulose is a widely used natural polymer that is safe, eco-friendly, and abundant in nature. It can be used as a matrix or reinforcing element in foam production [8].

Xanthan gum is an extracellular polysaccharide produced by the microorganism *Xanthomonas campestris*, which is commercially produced through fermentation [9]. It is soluble in cold water and is commonly used in the food, cosmetic, and pharmaceutical industries [10]. Although there are limited studies on foam materials produced from xanthan gum, previous research has focused on improving foam dispersion and foaming properties [11], producing foam materials for biomedical applications [12] and phase-change materials [13] and stabilizing dermal foam [14]. However, there have been no studies on using xanthan gum in the packaging and construction sectors.

Chitosan is a commonly used material for foam production but has some drawbacks. Firstly, it can cause corrosion in metal tools due to its ability to dissolve in acidic environments. Secondly, creating the right conditions for chitosan dissolution can be time-consuming. Similarly, starch requires specific conditions, such as cooking at a certain temperature, pressure, and mixing speed, for foam production. In contrast, xanthan gum can be dissolved directly and quickly in water, making it a more efficient option for foam production. Foam made from xanthan gum can be produced in a shorter time than foam made from other biopolymers. Xanthan gum’s properties are superior to other biopolymers for foam material production, such as its solubility in cold water, its ability to form gels with a high viscosity and elasticity, and its resistance to heat, pH, and enzymes [15]. On the other hand, there may be drawbacks or challenges associated with using xanthan gum for foam production. These could include the cost of xanthan gum production, the scalability of its manufacturing process, and the mechanical properties of the foams produced from xanthan gum. In particular, xanthan gum is utilized in commerce as the second microbial polysaccharide after dextran. Due to its wonderful qualities, including its non-toxicity, its capacity for cell proliferation, and its non-sensitizing effect, xanthan gum is regarded as an essential natural and industrial biopolymer. The side chains of xanthan gum can be folded down to the backbone by increasing the ionic strength, which results in a more ordered and stable structure with a high glass transition temperature. Its helical structure exhibits an organized shape, which prevents xanthan gum molecules from depolymerization; xanthan gum has more durable thermal behavior against hydrolysis than other water-soluble polysaccharides [16]. It also enables the production of foam material easily and quickly. For all these reasons, xanthan gum is particularly suitable for this study.

Optimizing the production of foam materials requires understanding the impact of polymer quantities on their properties. However, this process can be time-consuming and costly. To estimate output values efficiently, a sufficient number of experimental results must be considered. Industries aim to find optimal production conditions to minimize material and product losses. However, testing numerous mixture recipes can be time-, energy-, and cost-intensive. Therefore, economical methods that leverage technology and computer-based programs are needed to achieve the desired physical and mechanical properties of foam materials.

Machine learning is a discipline that holds great importance in many application areas today, and it has been successfully used to solve problems such as data analytics, prediction, and classification. Analyzing materials and composites uses various methods such as multiple linear regression, support vector machines, artificial neural networks, and least squares methods for different purposes. These techniques have been utilized to predict screw pull-out strengths and flexural modulus values of particleboards [17,18], to classify alloys based on their shapes [19], to predict sound absorption coefficients of sandwich-structured materials [20], to detect protozoa in wastewater [21], to model the strength of lightweight foam concrete [22], and to determine the processing parameters for drilling glass-laminated aluminum-reinforced epoxy composites [23]. However, no studies on machine learning related to xanthan-gum-based foam materials exist.

Machine learning is a significant discipline that finds extensive applications across various fields. It has demonstrated remarkable effectiveness in addressing challenges such as data analytics, prediction, and classification. Within the materials and composites domain, diverse methodologies, including multiple linear regression, support vector machines, artificial neural networks, and least squares methods, have been carried out for various purposes. These techniques have provided successful outcomes in predicting screw pull-out strengths and flexural modulus values of particleboards [17,18], classifying alloys based on their shapes [19], forecasting sound absorption coefficients of sandwich-structured materials [20], detecting protozoa in wastewater [21], modeling the strength of lightweight foam concrete [22], determining processing parameters for drilling glass-laminated aluminum-reinforced epoxy composites [23], estimating performance in different applications [24] and organic photovoltaic design [25]. Additionally, in the context of predicting material properties and minimizing both material waste and cost losses, machine learning methods provide considerable advantages.

In this study, the effects of different ratios of xanthan gum and cellulose fibers, produced in a 5% citric acid medium, on the density, compression modulus, and flexural modulus of foams were investigated in the first stage. In the second stage, using the values obtained from the experimental study, models were designed based on five machine-learning-based methods to predict the best results for the density, compression modulus, and flexural modulus, aiming to obtain more economical and reliable results without extensive testing.

## 2. Material and Methods

### 2.1. Materials

Cellulose was acquired from EUROPAP in Izmir, Turkey, and was produced from Eastern Larch with a density of 0.55 g/cm^3^. Citric acid with a density of 1.65 g/cm^3^ was purchased from Kimetsan Chemical in Ankara, Turkey. Xanthan gum, which has a density of 1.48 g/cm^3^, and sodium dodecyl sulphate (SDS) were obtained from Aromel Chemical in Konya, Turkey.

### 2.2. Production Process of Foams

The foam material was produced via a three-step process involving mixing, cross-linking, and foaming. The composition of the foam is given in Table 1. In the first step, 5% cellulose was mixed with 5% citric acid in a beaker using a mechanical stirrer at 600 rpm for 20 min. The citric acid acted as a cross-linking agent to enhance the mechanical strength of the foam, as reported by previous studies [26].

**Figure 1:**
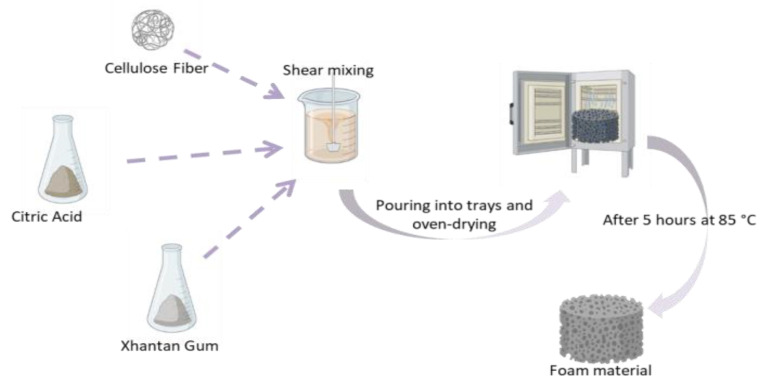
Production of foam produced from biopolymers.

The pH of the mixture was measured to be 4.2 using a pH meter. In the second step, xanthan gum was added to the mixture at different concentrations (2%, 5%, or 8%) and stirred at 1000 rpm for 60 min to form a homogeneous gel. In the third step, 0.1% SDS was added to the gel and the mixture was stirred at 3000 rpm for 15 min to create bubbles and aerate the gel. The resulting foam suspension was poured into aluminum trays and dried in a Goldterm brand dry air sterilizer (Istanbul/Turkey) at 85 °C for 5 h. The dried foam samples were labeled XCF-1 to XCF-5 according to their xanthan gum and cellulose contents. The production process of the foam material is shown in Figure 1.

### 2.3. Characterization of Foams

The foam specimens were conditioned for 24 h at a temperature of 20 °C and a relative humidity of 65% before conducting the tests. The density of the foam was determined in accordance with the ASTM C303 (2010) standard [27]. Compression and flexural modulus tests were performed using a Marestek universal testing machine (Mares Engineering Research Electronic Systems, Istanbul, Turkey) in accordance with the ASTM C165-07 (2017) [28] and ASTM C203-05a (2012) standards [29], respectively. A scanning electron microscope (Jeol brand and JSM-7600F model, Tokyo, Japan) (SEM) was used to analyze the microstructures of the foams. A layer of gold was applied to the surfaces of the foams before the examination to improve conductivity. The SEM was operated at an imaging voltage of 15 kV to observe the microstructure.

### 2.4. Machine-Learning-Based Prediction Methods of Foam Properties

**Figure 2:**
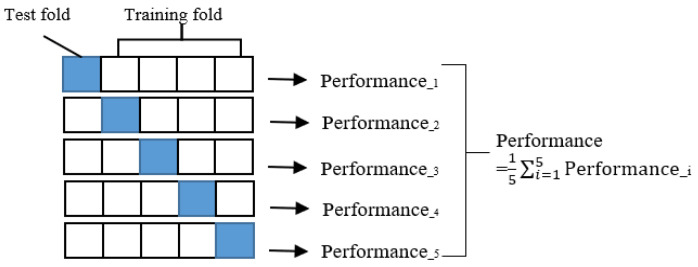
Five-fold cross-validation.

To compare the predictive performance of different machine learning methods for foam formulation components, we used k-fold cross-validation as an evaluation technique. K-fold cross-validation is a resampling method that splits the original data into k equal-sized subsets or folds, and then uses one fold as the test set and the remaining k − 1 folds as the training set [30]. This process is repeated k times, each time using a different fold as the test set, and the average of the k test results is used as the overall performance measure [31]. K-fold cross-validation has several advantages over using a single train/test split: It reduces the variance in the performance estimate, since it uses multiple test sets instead of one [32]. It prevents overfitting, since the model is tested on unseen data in each iteration [33]. It utilizes all the data for both training and testing, which is beneficial when the data are limited. It provides insight into how the model generalizes to new data, which is important for practical applications [34]. We chose five-fold cross-validation for this study, meaning that we divided the data into five folds and repeated the process five times [35]. Figure 2 illustrates this procedure.

The *k* results can be averaged (or otherwise combined) to produce a single estimate [36].

The error in guessing the *k* th part is:

(1) $$E_{k}\left(\gamma \right)=\sum_{i\in k\;th\;part}{\left(y_{i}-x_{i}{\hat{\alpha }}^{-k}(\gamma )\right)}^{2}$$

The cross-validation error is:

(2) $$CV\left(\gamma \right)=\frac{1}{k}\sum_{i=1}^{k}E_{i}(\gamma )$$

We used this error metric to compare and select the best machine learning model for our study. Cross-validation is a widely used and reliable technique for assessing model performance, especially when dealing with data uncertainties and overfitting issues [37,38].

#### 2.4.1. Multiple Linear Regression (MLR)

MLR is a statistical method that examines the relationship between a dependent variable and one or more independent variables. This regression method allows for the analysis of the relationship between the output variable, denoted as y, and a set of input parameters, represented as x_1_, x_2_, …, x_n_. The relationship between these independent and dependent variables can be linear or curvilinear. Independent variables are selected, and a mathematical model is developed based on the data that explain their connection with the dependent variable. This model is used to find the estimated value of the dependent variable [39,40]. When a model is used that includes multiple independent variables, its formulation is as follows:

y = α + *β*_1_x_1_ + *β*_2_x_2_ + *β*_3_x_3_ + ε (3)

where *y* is the predicted variable; x_1_, x_2_, and x_3_ represent the independent variables; *β* values represent the beta values corresponding to these *x* values; and ε is the error in the observed value.

#### 2.4.2. Least Squares Method (LSM)

The LSM is a standard regression method used to write the mathematical relationship between two physically interconnected variables as an equation that is as accurate as possible. The basis of the LSM is to find values that will minimize the sum of the squares of the total deviations [41].

For this method, first, data averages are found, and these average values create a point on the line of best fit. Then, the distance of each point to the line is calculated, and the sum of the squares of these distances is minimized. Mathematically, the LSM uses two equations to find the a and b coefficients:

(4) $$b=(\sum (x_{i}-\bar{x})(y_{i}-\bar{y}))/(\sum {\left(x_{i}-\bar{x}\right)}^{2})\;and\;a=\bar{y}-b\bar{x}$$

where $\bar{x}$ and $\bar{y}$ are the means of the independent and dependent variables, respectively.

#### 2.4.3. Support Vector Machine (SVM)

SVM is a machine learning tool defined by Vapnik in 1992. It is considered a non-parametric method based on kernel functions and is used for classification and regression. SVMs are an effective classification tool, especially for small- and medium-sized nonlinear datasets [42].

This study used ε-SVM regression to model the relationship between the data using linear or nonlinear functions. This method ignores errors within the ε distance from the observed value and sets the loss function based on the distance between the observed value and the ε boundary. Support vectors are data points with errors greater than ε, and SVM uses a function dependent only on support vectors to predict new values.

In ε-SVM regression, the training dataset includes predictor variables and observed response values. The goal is to find a function *f*(*x*) for each training point *x*, which deviates from *y_n_* by a value no greater than *ε* and is as flat as possible.

(5) $$\hat{y}=f\left(x\right)=w^{T}\emptyset \left(x\right)+b$$

The coefficient *w* is a one-dimensional array, and the superscript “*T*” denotes transpose. ∅(*x*) is a nonlinear transformation function used to map the input space to a higher-dimensional feature space.

$\left|y_{i}-{\hat{y}}_{i}\right|$ is the *ε*-insensitive loss function:

(6) $${\left|y_{i}-{\hat{y}}_{i}\right|}_{\varepsilon }=\left\{\begin{array}{cc} 0 & if\left|y_{i}-{\hat{y}}_{i}\right|\leq \varepsilon \\ \left|y_{i}-{\hat{y}}_{i}\right|-\varepsilon , & otherwise \end{array}\right\}$$

The cost function *Jε*(*w*, *ξ*, ξ*) defined in Equation (7) is minimized to find the values of w and b that satisfy the constraints.

(7) $$J\left(w,\xi ,\xi_{i}^{*}\right)=\frac{1}{2}w^{T}w+C\sum_{İ=1}^{N}(\xi +\xi_{i}^{*})\;$$

The constraints are given by $y_{i}-{\hat{y}}_{i}\leq \varepsilon +\xi ,\;{-y}_{i}+{\hat{y}}_{i}\leq \varepsilon +\xi_{i}^{*},\;\xi \geq 0,\;\xi_{i}^{*}\geq 0,\;İ=1,\;2,\ldots ,\;N.$ *ξ_i_* and $\xi_{i}^{*}$ are measures of positive errors, and *C* is a constraining constant. 

*ξ* and ξ* are slack variables used to calculate the classification errors of data points. ξi measures how far the *i*-th example data point is from the decision boundary, while ξ* measures how far the support vectors are from the decision boundary. The constant *C* controls the penalty applied to observations that fall outside the epsilon margin (*ε*) and prevents overfitting (regularization). This value determines the balance between the flatness of *f*(*x*) and the amount of tolerance for deviations larger than *ε* [43].

#### 2.4.4. Artificial Neural Networks (ANNs)

An ANN is a mathematical model inspired by the human brain’s biological neural network. It consists of units called neurons, which are organized into different layers, such as input, hidden, and output layers. The outputs of an ANN depend on the number of neurons in the hidden layer, activation function, and learning algorithm. These parameters vary depending on the number of input and output data. The process of generating outputs involves summing the weighted input signals, adding a bias, passing through an activation function, and producing the outputs, as expressed by Equation (8) [44]:

(8) $$y=\varphi (b+\sum_{i=0}^{m}x_{i}w_{i})$$

where *x_i_* is the input value, *w_i_* is the weight, *m* is the number of data samples, *b* is the bias, and *φ* is the activation function.

The parameters of the model, the weights, and the biases are initialized randomly before the training of the model and are optimized during the training process. The training process is where the model optimizes its parameters to accurately predict the target outputs. This is achieved by utilizing a loss function, which measures the disparity between the model’s predictions and the actual results and aims to minimize this discrepancy. During training, the examples in the dataset are given to the input layer of the model, and then the predictions in the output layer are calculated. These predictions are compared to the real results to calculate the value of the loss function. Then, the gradient of the loss function for the parameters is computed using the back-propagation algorithm, and the parameters are updated in the opposite direction of this gradient. This process is repeated for several epochs or until the loss function drops below a certain threshold.

#### 2.4.5. Generalized Regression Neural Networks (GRNNs)

A GRNN is a type of radial basis function neural network introduced by Specht [45]. The GRNN architecture has a radial basis layer and a special linear layer. It is similar to a radial basis network, but has a slightly different second layer with an additional linear layer before the output layer. The role of this layer is to calculate the regression from the data in the previous layer. The weight of the linear layer is trainable, but there is no bias in the linear layer. 

The GRNN model directly approximates any random function between the input and output vectors and uses a one-way learning algorithm instead of a repetitive learning algorithm like back-propagation (BP) [46]. The one-way learning algorithm is more efficient in terms of time and computational power by training all network layers at once. Previous studies have demonstrated that GRNN models, when trained with substantial amounts of input data, exhibit lower errors compared to other variants of artificial neural networks (ANNs) [47]. The GRNN model is based on kernel regression [48].

$$a^{1}=radbas(\|w-p\|b)\;\;\;\;a^{2}=purelin(n^{2})$$

The spreading parameter determines how wide an area the GRNN network will cover. It determines the width of the radial basis functions [49]. The relationship between the bias, *b*, and the spread of the Radbas function is given by:

(9) $$b=\frac{0.8326}{spread}$$

The spreading parameter has a significant impact on the performance of the network. It determines the range of influence that each data point will have on the network and, thus, affects the overall performance of the network. Proper selection of the spreading parameter is crucial for ensuring that the network operates effectively and produces accurate predictions [50].

## 3. Results and Discussion

### 3.1. Effect of Components on the Physical and Mechanical Properties of Foams

The effects of citric acid, cellulose fiber, and xanthan gum on the density, compression modulus, and flexural modulus of foam materials were investigated. Table 2 presents the mean densities, compression modulus, and flexural modulus of foam materials containing different ratios of xanthan gum and cellulose fibers.

It has been found that the density of foams used in production varies between 49.42 kg/m^3^ and 172.71 kg/m^3^, depending on the concentration of the materials used. The porous structure of the produced foams allows them to dry without any structural collapse and enables the production of lightweight materials. The clustering of cellulose fibers occurs due to attractive forces. Removing the solvent in the suspension also causes the xanthan gum and cellulose fibers to intertwine. It is known that this has a significant effect on the density increase of the produced foam material [51]. Xanthan gum has the most significant effect on the density value of the produced foam material. The density values of produced foam materials vary over a wide range. Because the amount of polymers used increases, the solid content within the final foam material also increases. As a result, the densities of foam materials increase, leading to different values. Furthermore, xanthan gum has the greatest impact on density. This is due to xanthan gum having a density of 1.48 g/cm^3^, while cellulose has a density of 0.55 g/cm^3^. Therefore, even if these polymers are present in the foam material in equal proportions, xanthan gum increases the density of the produced foam material to a greater extent. It has been stated that foam materials produced with similar biopolymers also exhibit a wide range of density values [4,11,51]. This situation explains the reason behind the wide range of density values in foam materials. The produced foams are of a density that can be evaluated in areas such as insulation, cushioning, and packaging. The density of biodegradable and synthetic foams for different purposes ranges from 5 kg/m^3^ to 930 kg/m^3^ [52,53,54]. The density of aerogels produced from xanthan gum, clay, and agar via the freeze-drying technique ranges from 44 kg/m^3^ to 101 kg/m^3^ [55]. Surfactants such as SDS are added to reduce the density value in biopolymer-based foam production [56].

**Figure 3:**
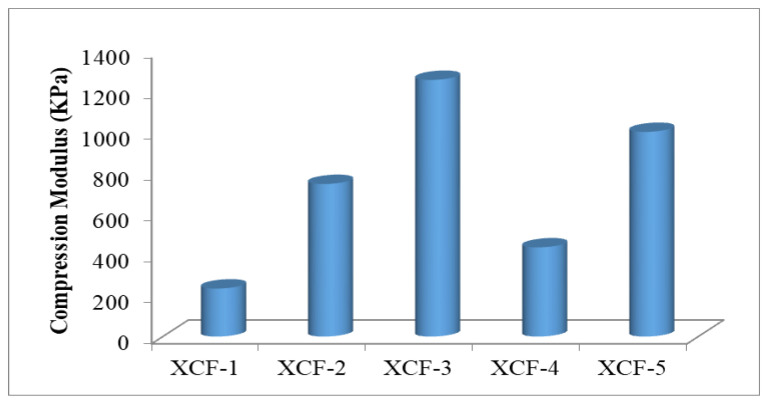
The compression moduli of cellulose-reinforced xanthan-gum-based foams.

The compression modulus values of foams are important because they demonstrate the ability of the foam to resist compression and deformation under an applied load. A higher compression modulus value implies that the foam is more resistant to compression or deformation and can better maintain its shape and structure under pressure. The compression modulus values of foams are significant in applications where the foam is used as a cushioning or support material, such as in mattresses or furniture. Foams with higher compression modulus values can provide more significant support and maintain their shape over time, improving comfort and durability. Moreover, the compression modulus values of foams can also impact their performance in other applications, such as in packaging or insulation materials. Foams with higher compression modulus values may be more suitable for packaging applications, as they can better protect the contents of the package from external forces. In comparison, foams with lower compression modulus values may be better suited for insulation applications [57]. The compression modulus values of cellulose-reinforced xanthan-gum-based foams are given in Figure 3.

As shown in Figure 3, the compression modulus values of xanthan-based foam materials ranged from 235.25 KPa to 1257.52 KPa. XCF-3 showed the highest compression modulus value. The lowest compression modulus value of 235.25 KPa was obtained from XCF-1. The compression modulus is dependent mainly on the density of the foams. As the density of the material increases, the force applied per unit area decreases, thus increasing the compression modulus.

Additionally, this improvement in compression modulus is likely due to the polymer network presented in XCF-3, which provides a more efficient stress distribution than other samples and prevents early structural failure of the foam structure due to buckling or fracture [55]. In previous studies, the compression modulus of starch-, chitosan-, and cellulose-based foams with different densities ranged from 13 KPa to 12,500 KPa [4,58,59]. The compression modulus of foam produced by reinforcing xanthan gum with agar and clay ranged from 600 KPa to 4770 KPa [55]. Commercially available foam materials such as EPS and PU foams have a wide range of mechanical properties, with compression moduli ranging from 2 to 48,000 KPa [60]. Based on all of these results, the produced foam material is within an acceptable range.

**Figure 4:**
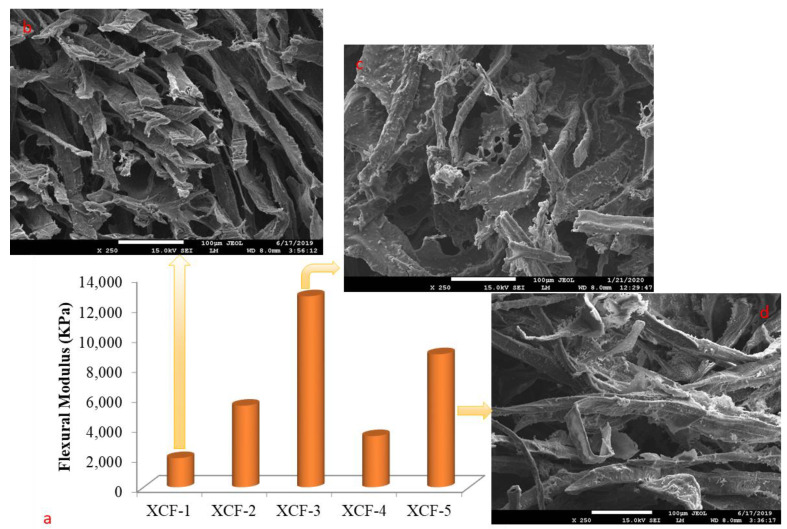
(**a**) The flexural modulus values of cellulose-reinforced xanthan-gum-based foams. SEM images of (**b**) XCF-1, (**c**) XCF-3, and (**d**) XCF-5 at ×250 magnification.

The flexural modulus values of foams indicate the stiffness and rigidity of the foam when it is subjected to a bending force. A higher flexural modulus value implies the foam is stiffer and more resistant to bending or deformation. The flexural modulus values of foams are significant in applications where the foam is used as a structural material, such as in construction or automotive industries [61]. Foams with higher flexural modulus values can provide greater structural support and better withstand external forces, such as weight or impact. In addition, the flexural modulus values of foams can also impact their performance in other applications, such as in cushioning or packaging materials. Foams with lower flexural modulus values may be more suitable for cushioning applications, as they can absorb more energy upon impact, while foams with higher flexural modulus values may be better suited for packaging applications, as they are able to provide more structural support and better protect the contents of the package [62]. The flexural modulus values and SEM images of cellulose-reinforced xanthan-gum-based foams are given in Figure 4.

The flexural modulus values vary between 1939.76 KPa and 12,736.39 KPa, depending on the concentration (Figure 4a). The highest flexural modulus value was obtained from XCF-3, which contains 8% xanthan gum. As the quantity of components used in production increased, the flexural modulus also increased with the density values of the produced material. Raymond and Rodrigue (2013) also found similar results in their study [63]. In addition, it was found that xanthan gum had a more significant effect on flexural modulus values than cellulose. Xanthan gum, used as a matrix, is considered to contribute to forming the cell wall in the foam material, as shown in Figure 4c. Cellulose fibers interact with the matrix through carboxyl or hydroxyl groups (Figure 4d), creating a more compatible interface that leads to stress transfer, resulting in an increase in the flexural modulus [64]. Yildirim (2018) found a flexural modulus of 15,082 KPa in the foam material produced, which is quite close to the results in the current study [65]. The difference may be due to the matrix material used.

When xanthan gum and cellulose are mixed together, they can interact physically and chemically. Physically, they can form a composite material that has both the properties of the individual components and some new properties that emerge from their interaction. The xanthan gum network can act as a binder that holds the cellulose fibers together, while the cellulose fibers can reinforce the xanthan gum network and prevent it from collapsing during the drying process [51]. Chemically, hydrogen bonds can form between the hydroxyl groups of xanthan gum and cellulose. Cellulose can interact strongly with xanthan gum due to the increased electrostatic attraction between the cellulose, which has a carboxyl group at the C_6_ position, and the glucuronic acid groups of xanthan gum [66]. Also, xanthan gum has the greatest impact on mechanical properties due to its role as the matrix in the produced foam material. This is because the thicker the cell walls of the foam material, the more resistance it can provide against mechanical effects. Therefore, as the amount of each component increases, the density also increases, resulting in better mechanical properties of foams due to a decrease in the load per unit area [67]. In cellulose-based materials, such as foam and paper, the primary source of resistance to mechanical stress occurs predominantly from the points of contact between individual fibers [68]. Cellulose fibers improve the mechanical resistance of these contact points through polymer entanglements and increase the interfacial contact area [60]. The hydroxyl groups present in cellulose allow for polar–polar and hydrogen bond interactions, improving the mechanical properties of the produced foams [69]. Additionally, it has been reported that an increase in the concentration of citric acid by up to 5% can increase the crosslinking degree between cellulose macromolecular chains and allow for the formation of additional hydrogen bonds, resulting in a highly linear structure that enhances the mechanical properties of foam materials [70]. Previous studies have shown that foam materials produced with the addition of 5% citric acid exhibited an increase in their mechanical properties ranging from 30% to 37% [71,72]. Hence, there is a close relationship between the density of foam materials and the compression and flexural moduli in this study. This is stated as one of the reasons for the wide range of mechanical properties exhibited by foam materials.

### 3.2. Machine-Learning-Based Prediction of Foam Properties

**Figure 5:**
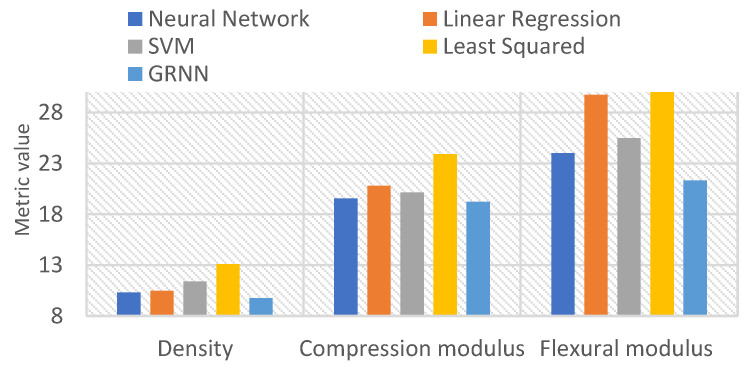
Comparison of average RMSE values of models on test data.

In this research study, a dataset consisting of 45 distinct experimental samples was employed to predict various parameters such as density, compression modulus, and flexural modulus. To assess the performance of the models, a generalized regression neural network (GRNN), support vector machines (SVMs), an artificial neural network (NN), multiple linear regression (MLR), and least squares methods were used. A rigorous evaluation of these models was conducted using the k-fold cross-validation method (k = 5), which allowed for reliable predictions despite the constraints imposed by the modest dataset. Five different performance measures were measured for each model, and the average of these measures was used to determine the model performance. The mean squared error (MSE) and R-squared measurements obtained during training for each method are presented in Table 3 for each parameter. Furthermore, a performance comparison of the models on the test data is illustrated in Figure 5.

Table 3 presents the mean squared error (MSE) and R-squared values obtained during the training phase for the methods employed in predicting the density, compression modulus, and flexural modulus parameters. The MSE quantifies the deviation between the predicted and actual values, while the R-squared measures the goodness-of-fit of the model to the data. In Figure 5, the root mean squared error (RMSE) values of the methods are compared on the test data. To enhance clarity, the RMSE values for different parameters have been multiplied by specific factors (0.2 for compression modulus and 0.025 for flexural modulus).

Figure 5 compares the average performance of the models on test data obtained from cross-validation. The average RMSE values for each method were calculated for the density, compression, and flexural moment parameters. The GRNN method, which had the lowest RMSE value, performed best for all parameters. Multiple regression analysis is commonly used to develop a causal relationship between variables. The relationship between the dependent variable and multiple independent variables is expressed with a linear function. In this study, linear regression provided good results for density estimations. However, multiple regression analysis may not be suitable for solving some problems and may not perform well on data with complex structures. The least squares method had the highest RMSE values and provided the worst prediction results. SVM uses a linear or nonlinear function to model the relationship between data. Epsilon and C penalty values were automatically determined in all three models. SVM provided better results than the ANN.

The ANN model stands out because it can predict all outputs simultaneously, compared to models where separate models are obtained for each output. This study evaluated each prediction result designed as multiple outputs separately. The number of neurons in the hidden layer was set to 10 and it was trained using the Levenberg–Marquardt back-propagation algorithm.

**Figure 6:**
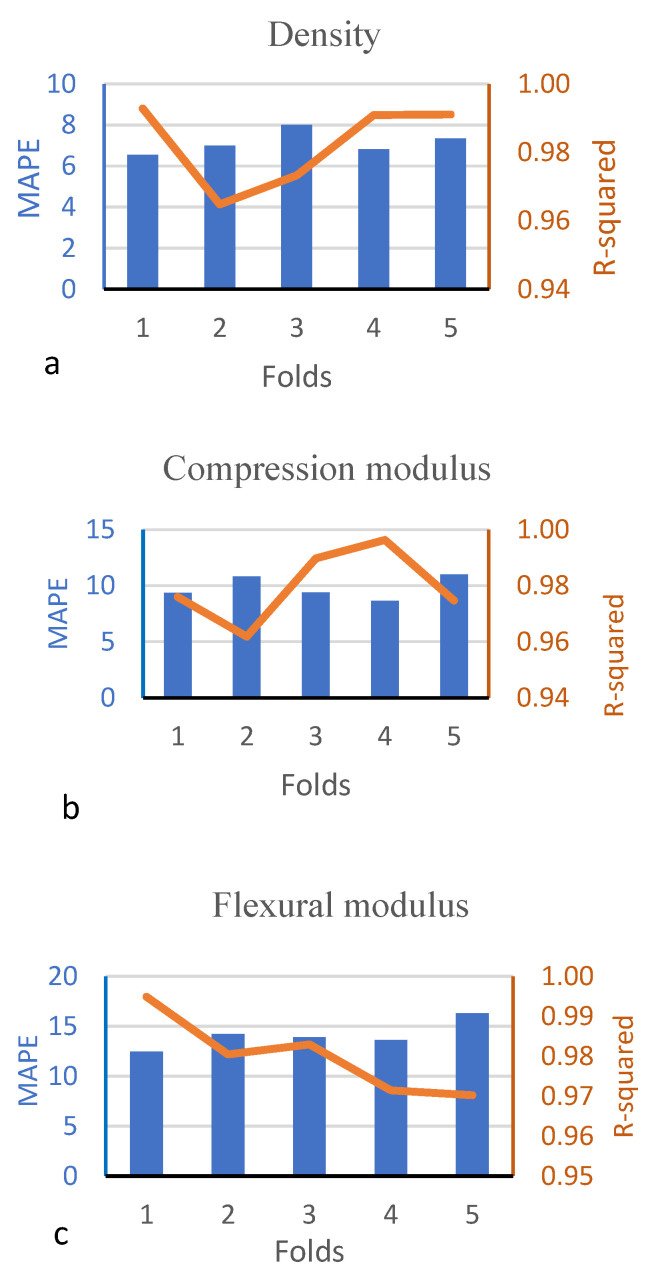
MAPE and R-squared test data values for five-fold cross-validation of the GRNN model. (**a**) Density, (**b**) compression modulus, (**c**) flexural modulus.

The GRNN model was designed with three inputs and three outputs. The spread parameter was determined as 0.7 by conducting experiments because it significantly impacts the performance of the GRNN model. As the spread parameter becomes too large, the accuracy level decreases significantly [49]. The mean absolute percentage error (MAPE) and R-squared test data values for five-fold cross-validation of the GRNN model in the test phase are shown in Figure 6.

In Figure 6, the MAPE and R-squared values of the test data in five-fold cross-validation for the GRNN model are shown for each fold separately. The MAPE is the average of the absolute errors as a percentage of the true values. Models with MAPE values below 10% are considered to have an outstanding performance, while those with MAPE values between 10% and 20% are considered good, models with MAPE values between 20% and 50% are considered acceptable, and those with MAPE values above 50% are considered incorrect and faulty [73]. Unlike the MAPE and R-squared, the MSE is scale-dependent and more sensitive to outliers since errors are squared [34]. Across different folds, it was observed that the MAPE ranged from 5% to 21%, and R-squared values ranged from 0.97 to 0.99.

The model developed with a GRNN has average MAPE values of 9.6%, 12.4%, and 16.4% for density, compression, and flexure predictions, respectively, and R-squared values of 0.97 and 0.99, indicating that this model is a good model. The total R-squared value of the model developed with a GRNN is 98%, and the MAPE value is 12.8%. When the performance of the regression models was examined, it was seen that the R-squared value is between 0.960 and 0.971. In the model created with a GRNN, the R-squared value ranges from 0.97 to 0.99, which is very close to 1.

Multi-output models like GRNNs have the potential to obtain more comprehensive results by modeling the relationships between multiple output variables accurately. This allows for better modeling of nonlinear relationships in the data structure. However, single-output ANNs are generally preferred in regression analysis. The abilities of NN models to predict many variables at the same time and to model nonlinear relationships with the data structure are some of their important advantages.

## 4. Conclusions

This study consists of two parts. In the first part, the density and mechanical properties of foams produced from xanthan gum and cellulose fibers were investigated. It was observed that the density of the foam could be controlled by adjusting the concentration of xanthan gum and the solid content in the foam material. The addition of xanthan gum had a significant effect on the density and mechanical properties of the foams, with higher concentrations leading to an increased density and improved compression and flexural modulus values. The cellulose fibers in the foams contributed to the mechanical resistance through polymer entanglements and increased the interfacial contact area. In the second part, ML models were trained using measured data, and the performance of these models was evaluated using cross-validation. The results showed that the GRNN method performed best, but SVM and NN methods also provided acceptable results. Evaluation methods such as cross-validation help to objectively evaluate the overall model performance. The GRNN model showed a high accuracy and a good performance, with R^2^ values above 0.97 and MAPE values below 16.4%. Considering the cost and time spent on experiments, using ML learning methods for predicting these values is important due to the demonstrated contribution to reducing the number of experiments. Xanthan gum foams can be utilized for indoor insulation purposes, cushioning, and packaging. The solubility of xanthan gum in external environments limits its usage. To enhance its water resistance and mechanical properties for application in external environments and the construction industry, cross-linking agents or hydrophobic polymers should be added during foam production. Additionally, for the produced foam to be applicable in biomedical fields, it could be used in combination with biopolymers such as chitosan, which possesses antibacterial properties. Numerous parameters influence the mechanical properties of foam materials. In future studies, new models can be developed by utilizing different parameters, such as varying mixture ratios and different types and quantities of polymers. Predicting the various technological and mechanical characteristics of foam materials can contribute to reducing costs in industry. 

## Figures and Tables

**Table 1:** Composition of the foam.

Codes	Xanthan Gum (%)	Citric Acid (%)	Cellulose (%)	SDS (%)
XCF-1	2	5	5	0.1
XCF-2	5	5	5	0.1
XCF-3	8	5	5	0.1
XCF-4	5	5	2	0.1
XCF-5	5	5	8	0.1

XCF: xanthan gum–cellulose foam.

**Table 2:** Mean density, compressive modulus, and flexural modulus values of xanthan-gum-based foams.

Codes	Density (kg/m^3^)	Compression Modulus (KPa)	Flexural Modulus (KPa)
XCF-1	49.42	235.25	1939.76
XCF-2	112.12	747.39	5436.99
XCF-3	172.71	1257.52	12,736.39
XCF-4	76.90	436.85	3408.63
XCF-5	137.52	1002.69	8869.27

XCF: xanthan gum–cellulose foam.

**Table 3:** R^2^ and MSE values of the methods for predicting density, compression modulus, and flexural modulus parameters.

	Density		Compression Modulus		Flexural Modulus	
	MSE	R^2^	MSE	R^2^	MSE	R^2^
Neural Network	69,195	0.9642	7031.36	0.9505	791,205	0.9501
Linear Regression	74,763	0.9613	7271.33	0.9489	1,373,913	0.9473
SVM	75,326	0.9478	7624.01	0.9463	834,695	0.9135
Least Squares Method	14,217	0.9398	12,971.6	0.9094	1,467,168	0.9074
GRNN	68,582	0.9820	7100.18	0.9749	796,872	0.9747

## Data Availability

Data are contained within the article.
